# Clinical Spectrum of Cutaneous, Ocular, and Hair Manifestations in Patients With Inborn Errors of Immunity: Insights From a Single Center in Turkey

**DOI:** 10.1002/iid3.70384

**Published:** 2026-02-25

**Authors:** Burcu Cil Yılmaz, Sibel Kaplan Sarikavak, Sezin Naiboglu, Gulsah Kalay, Erkan Cakmak, Ozge Turkyilmaz Ucar, Selami Ulas, Nermin Kapci, Pinar Gokmirza, Cigdem Aydogmus

**Affiliations:** ^1^ Department of Pediatric Allergy and Immunology University of Health Sciences, Basaksehir Cam and Sakura City Hospital Istanbul Turkey; ^2^ Present address: Department of Pediatric Allergy and Immunology, Haseki Training and Research Hospital University of Health Sciences Istanbul Turkey; ^3^ Present address: Department of Pediatric Allergy and Immunology, Gaziosmanpasa Training and Research Hospital University of Health Sciences Istanbul Turkey; ^4^ Present address: Department of Pediatric Allergy and Immunology, Tekirdag Ismail Fehmi Cumalioglu City Hospital University of Health Sciences Tekirdag Turkey

**Keywords:** cutaneous infections, disease‐specific skin manifestations, eczema, hair abnormalities, inborn errors of immunity, ocular manifestations, primary immunodeficiency

## Abstract

**Background:**

Inborn errors of immunity (IEI), previously referred to as primary immunodeficiencies, are a heterogeneous group of genetic disorders affecting immune development and function. While once considered rare, IEIs are increasingly recognized, particularly in regions with high consanguinity rates. Cutaneous manifestations, as well as ocular and hair abnormalities, may provide early and clinically relevant diagnostic clues. This study aimed to assess the prevalence, types, and diagnostic value of cutaneous, ocular and hair manifestations in patients with IEI.

**Methods:**

A total of 386 patients with confirmed IEI, classified according to the 2024 IUIS criteria, were retrospectively analyzed. Cutaneous, ocular (e.g., conjunctivitis, keratitis, scleral telangiectasia), and hair manifestations (e.g., alopecia areata, pigmentary abnormalities) were systematically reviewed from medical records. Skin findings were categorized as infectious, immune‐allergic (eczema, alopecia areata, urticaria, erythroderma), disease‐specific, or other.

**Results:**

Cutaneous, ocular, and/or hair manifestations were identified in 198 patients (51.3%), with 59.1% present at diagnosis. Infectious manifestations were the most common (71.8%), followed by immune‐allergic findings (34.8%), including eczema (30.3%), and disease‐specific manifestations (17.7%). Ocular findings were observed in 15.7% of patients, while hair abnormalities were present in 4.04%. Skin infections were predominantly bacterial (53.1%) and were most frequent in phagocytic and innate immunity defects. Eczema was most frequent in hyper‐IgE syndrome (85.8%), while non‐eczematous allergic findings were most common in immune dysregulation. Ocular involvement, including viral retinitis and scleral telangiectasia, and hair abnormalities, such as syndromic hair shaft defects and alopecia areata, were observed across multiple IEI subgroups.

**Conclusion:**

Cutaneous, ocular, and hair abnormalities are frequent in IEI and may support early diagnosis. Recognition of recurrent, atypical, or treatment‐resistant skin, eye, or hair findings should prompt immunological evaluation, particularly in pediatric patients.

## Summary

• Cutaneous, ocular, hair manifestations represent clinically meaningful and often early indicators of IEI.
• This study highlights the diagnostic value of infectious skin lesions, eczema, disease‐specific cutaneous findings, and selected ocular and hair abnormalities across different IEI categories.
• Integrating dermatologic and ophthalmologic assessment into routine clinical evaluation may facilitate earlier recognition of IEI and support timely immunologic work‐up and when indicated, molecular testing, particularly in pediatric populations.

## 1 Introduction

Inborn errors of immunity (IEIs), formerly referred to as primary immunodeficiency disorders, represent a heterogeneous group of genetic conditions marked by impaired immune system development or function. As outlined in the 2024 report by the International Union of Immunological Societies (IUIS), a total of 555 distinct IEI disorders have been classified to date, linked to pathogenic variants in 504 genes. This growing list includes disorders caused by both gain‐ and loss‐of‐function mutations, somatic mutations, and autoantibody‐mediated phenocopies [1].

Clinically, IEIs present with a broad spectrum of manifestations such as recurrent or severe infections, autoimmunity, autoinflammation, lymphoproliferation, allergy, bone marrow failure, and malignancy [1, 2]. Cutaneous findings are among the most common clinical features in inborn errors of immunity and often constitute the earliest manifestation of disease, particularly in children [3]. These may present as infectious skin lesions caused by bacterial, viral, or fungal agents, or as non‐infectious conditions including eczema, urticarial eruptions, granulomatous inflammation, and vasculitic changes [4]. Certain skin findings are strongly associated with specific IEI subtypes‐eczema in hyper‐IgE syndromes or granulomatous lesions in chronic granulomatous disease [5, 6].

Recognizing these dermatologic signs is critical for early diagnosis, genetic evaluation, and timely treatment of IEIs [7]. However, comprehensive data detailing the frequency and distribution of skin findings across IEI categories remain limited, particularly in consanguineous populations where these disorders are more common [8, 9].

This study aims to characterize the prevalence and spectrum of cutaneous, ocular, and hair manifestations in a large single‐center cohort and to evaluate their diagnostic relevance in the context of IEI.

## 2 Methods

**Figure 1:**
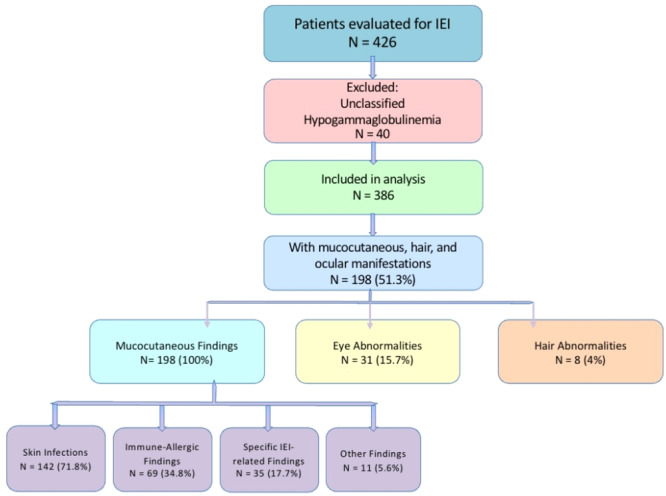
Flowchart of patient selection and categorization of mucocutaneous, ocular, and hair findings among individuals diagnosed with IEI. IEI, inborn errors of immunity.

A total of 426 patients diagnosed with inborn errors of immunity (IEI) at our center were retrospectively reviewed. Forty patients with unclassified hypogammaglobulinemia were excluded due to lack of definitive diagnosis. The remaining 386 patients were categorized according to the 2024 IUIS classification. Mucocutaneous, ocular, and hair manifestations were assessed and recorded. Cutaneous findings were further classified into four subtypes: infectious, immune‐allergic (including eczema, alopecia areata, urticaria, and erythroderma), specific IEI‐related lesions, and other findings. Fungal nail infections were classified under infectious cutaneous manifestations, whereas nail dystrophy observed in patients with ectodermal dysplasia was considered a disease‐specific manifestation. An overview of patient selection, classification, and distribution of clinical findings is provided in Figure 1.

### 2.1 Ethics Statement

The study was approved by the Clinical Research Ethics Committee of Başakşehir Çam and Sakura City Hospital (Protocol No: 2024‐95). Written informed consent was obtained from the parents or legal guardians of all patients included in the study. Additional consent was obtained for the publication of clinical photographs.

### 2.2 Statistics

Descriptive statistics were used to summarize the demographic and clinical characteristics of the patients. Categorical variables were presented as frequencies and percentages. To assess the relationship between skin manifestations and infections, Pearson correlation analysis was performed. A *p*‐value of less than 0.05 was considered statistically significant. Statistical analyses were performed using the SPSS software version 26.0 (IBM Corporation).

## 3 Results

**Figure 2:**
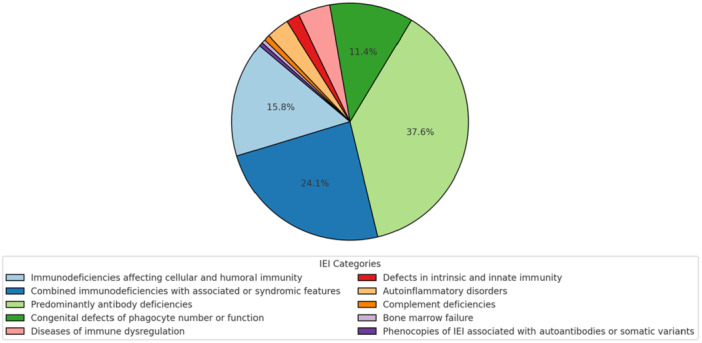
Distribution of inborn errors of immunity patients. IEI: inborn errors of immunity.

A total of 386 patients diagnosed with IEI, 198 (51.3%) exhibited cutaneous, ocular, or hair manifestations (the distribution of the entire IEI cohort according to the 2024 IUIS classification is shown in Figure 2). Within this group, 57.6% (*n* = 114) were male, and 59.1% (*n* = 117) presented with these findings at diagnosis, often contributing to the initiation of immunologic evaluation. A history of consanguinity was noted in 62.1% (n = 123) of patients with mucocutaneous, ocular, and/or hair involvement, suggesting a potential genetic contribution. Notably, 15.2% (*n* = 30) had more than one type of skin manifestation.

**Figure 3:**
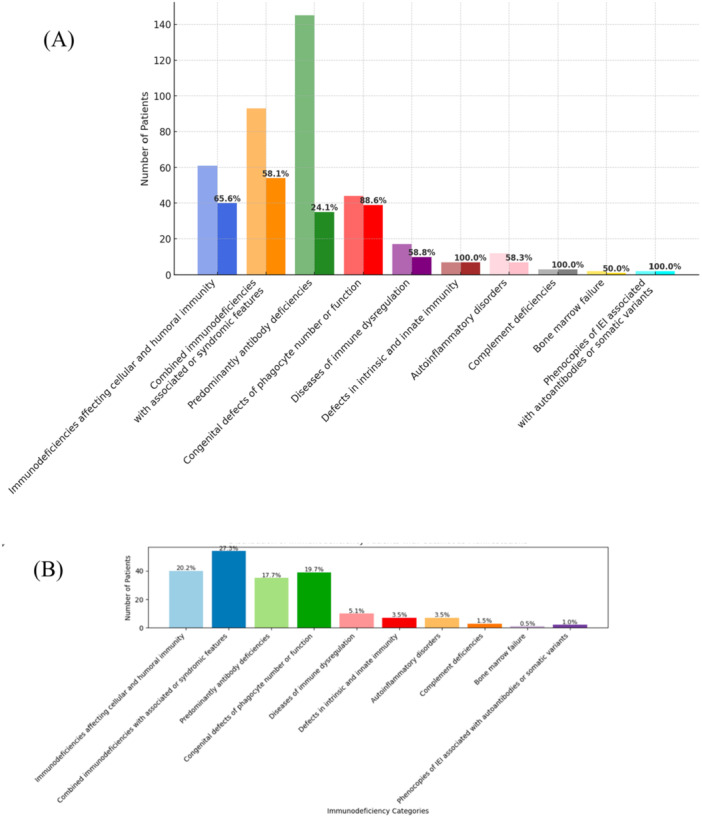
Distribution and proportion of mucocutaneous, ocular and hair manifestations across IEI categories. (A) Number of patients in each IEI category and the proportion of patients with cutaneous, ocular and hair manifestations (in %). (B) Distribution of IEI groups among the 198 patients who exhibited cutaneous, ocular and hair manifestations. These bar charts illustrate the relative frequency and representation of skin involvement across immunodeficiency categories, both in the total cohort (A) and within the subgroup of patients with skin findings (B).

The highest rates of mucocutaneous, ocular, and/or hair manifestations were observed in intrinsic/innate immunity defects, complement deficiencies, and phenocopies (100%, *n* = 2/2), followed by phagocytic disorders (88.6%, *n* = 39/44) and immunodeficiencies affecting cellular and humoral immunity (65.6%, *n* = 40/61). Predominantly antibody deficiencies showed the lowest rate (24.1%, *n* = 35/145). Syndromic combined immunodeficiencies, immunodeficiencies affecting cellular and humoral immunity, and phagocytic disorders were the most represented groups among patients presenting with these manifestations (Figure 3A,B).

Within this group, patients with involvement of the cutaneous, ocular, and/or hair exhibited a broad and heterogeneous spectrum of genetically defined inborn errors of immunity. Among immunodeficiencies affecting cellular and humoral immunity, a substantial proportion of patients had severe combined immunodeficiency (SCID), most frequently associated with defects in RAG1/RAG2, IL2RG, JAK3, ADA, DCLRE1C (Artemis), and NHEJ1 (Cernunnos), while a small subset of patients underwent hematopoietic stem cell transplantation prior to molecular confirmation. Combined immunodeficiencies were most commonly linked to pathogenic variants in CD40LG and DOCK8, followed by MALT1, MHC class II genes, ARPC1B, FCHO1, and STK4. Among syndromic combined immunodeficiencies, DNA repair defects constituted the largest subgroup, predominantly represented by ATM deficiency. Thymic defects with congenital anomalies, mainly 22q11.2 deletion syndrome, and hyper‐IgE syndromes (HIES) associated with STAT3, PGM3, SPINK5, and complete IL6ST deficiency were also frequently observed. Additional syndromic disorders included anhidrotic ectodermal dysplasia with immunodeficiency (IKBKG), PNP deficiency, SP110‐related immunodeficiency, trichohepatoenteric syndrome (TTC37), and RBCK1‐associated immunodeficiency. Predominantly antibody deficiencies (PAD) accounted for a substantial proportion of affected patients, including agammaglobulinemia associated with BTK and IGLL1 defects, CVID phenotypes most frequently linked to TNFRSF13B and NFKB1, as well as selective or combined immunoglobulin isotype deficiencies. Disorders of phagocyte number or function were predominantly represented by congenital neutropenia, most frequently associated with HAX1 deficiency, followed by defects in SRP54, USB1, JAGN, and ELANE. Chronic granulomatous disease (CGD) constituted a substantial subgroup, most commonly associated with CYBB and NCF1 mutations, whereas leukocyte adhesion deficiency type 1 was less frequently observed. Diseases of immune dysregulation included defects in LYST, RAB27A, IL10R, AIRE, CASP10, FAS, LRBA, and TNFRSF9, while defects in intrinsic and innate immunity most commonly involved IL12RB1, STAT1, IRAK4, GATA2, and MYD88. Autoinflammatory disorders were predominantly associated with ADA2 deficiency, whereas complement deficiencies mainly involved SERPING1 mutations. One patient with bone marrow failure due to RTEL1 deficiency and two phenocopies with chronic mucocutaneous candidiasis (CMC) without molecular confirmation were also included. Detailed genetic data are provided in Supporting Information S1: Table S1.

The most common cutaneous findings were skin infections (71.8%, *n* = 142/198), followed by eczema (30.3%, *n* = 60/198) and immune‐allergic conditions overall (34.8%, *n* = 69/198). Non‐eczematous allergic findings (e.g., alopecia, urticaria) were observed in 4.5% (*n* = 9/198). Specific IEI‐related lesions were seen in 17.7% (*n* = 35/198), while ocular and hair abnormalities were present in 15.7% (*n* = 31/198) and 4.04% (*n* = 8/198), respectively.

**Figure 4:**
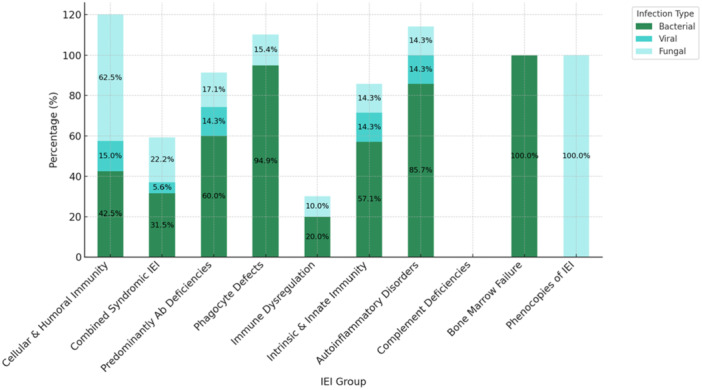
Distribution of bacterial, viral, and fungal cutaneous infections across IEI groups. Percentages are calculated based on patients with mucocutaneous, ocular, and hair findings in each category. (Ab, antibody; IEI, inborn errors of immunity).

Skin infections were detected in 142 patients (36.8% of the total cohort), with the highest frequencies in phagocytic defects (88.6%, *n* = 39/44), intrinsic/innate immunity defects (85.7%, *n* = 6/7), and autoinflammatory disorders (58.3%, *n* = 7/12). A strong positive correlation was observed between the presence of skin infections and the coexistence of additional non‐infectious cutaneous manifestations (*r* = 0.904, *p* = 0.0008). Bacterial infections were most common (53.1%, *n* = 105/198), followed by fungal (27.3%, *n* = 54/198) and viral infections (8.1%, *n* = 16/198). The distribution of infection types across IEI subgroups is presented in Figure 4. Bacterial infections were predominant in most groups, particularly in phagocyte defects (94.9%, *n* = 37/39), intrinsic and innate immunity defects (85.7%, *n* = 6/7), and autoinflammatory disorders (85.7%, *n* = 6/7). Fungal infections were most notable in patients with defects in cellular and humoral immunity (62.5%, *n* = 25/40) and combined immunodeficiencies (22.2%, *n* = 12/54). Although 100% (*n* = 2/2) of patients with phenocopies of IEI had fungal infections, this group comprised a very limited number of patients, warranting cautious interpretation. In addition, one patient with CGD developed invasive aspergillosis with cutaneous extension. Viral skin infections were relatively rare overall, but observed in up to 14.3% of patients in several categories, including autoinflammatory disorders, innate immunity defects, and antibody deficiencies. In our cohort, BCGitis occurred in 13.6% (*n* = 27/198) of patients, predominantly among those with SCID, CGD, and Mendelian susceptibility to mycobacterial disease (MSMD).

Eczema was observed in 15.5% of the total cohort (*n* = 60/386), most notably in HIES (85.8%, *n* = 6/7), and to a lesser extent in PAD and combined immunodeficiencies. Non‐eczematous allergic findings were more prominent in immune dysregulation syndromes. Hair and eye abnormalities were also most frequent in syndromic combined immunodeficiencies and immune dysregulation.

Nail involvement was not analyzed as a separate category. Fungal nail infections were therefore included among infectious cutaneous manifestations and were observed in patients with defects affecting cellular and humoral immunity and immune regulation, including ATM, STAT3, DOCK8, and AIRE deficiencies, as well as in phenocopies of IEI with CMC. In contrast, nail dystrophy observed in patients with ectodermal dysplasia with immunodeficiency was classified as a disease‐specific manifestation.

Ocular involvement was observed across several IEI subtypes and was predominantly associated with severe and combined immunodeficiencies and DNA repair defects. Viral retinitis accompanied by keratitis or conjunctivitis was documented in patients with SCID due to ADA and JAK3 deficiencies, as well as in combined immunodeficiency associated with ARPC1B deficiency. In addition, recurrent viral ocular involvement, including Epstein–Barr virus–associated retinitis and conjunctivitis, was observed in a patient with immune dysregulation due to TNFSF9 deficiency. Scleral telangiectasia represented the most frequent ocular finding and was predominantly observed in patients with ATM deficiency, occasionally accompanied by recurrent conjunctivitis.

Hair abnormalities were less frequent but showed high diagnostic specificity. Patients with trichohepatoenteric syndrome due to TTC37 deficiency exhibited characteristic hair shaft abnormalities. Silvery‐gray hair was observed in patients with Chediak–Higashi syndrome (LYST deficiency) and Griscelli syndrome (RAB27A deficiency). Autoimmune‐mediated alopecia areata was observed in a patient with immune dysregulation due to AIRE deficiency. Alopecia areata was also documented in a patient with PLCG2‐related autoinflammatory disease.

Detailed clinical findings across IEI subgroups—including cutaneous, ocular, and hair abnormalities—are summarized in Supporting Information S1: Table S2, while their phenotypic distribution across IEI categories is illustrated in Supporting Information S1: Figure S1 and S2.

**Figure 5:**
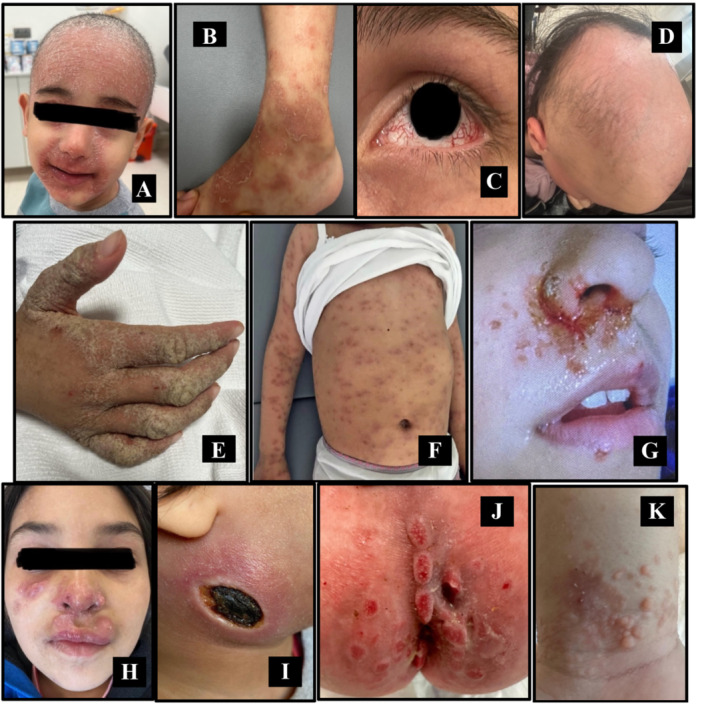
Representative clinical photographs of selected cutaneous, ocular, and hair manifestations observed in patients with specific IEIs (Panels A‐K). (A) Psoriasiform erythroderma in a patient with ataxia–telangiectasia (AT) due to functionally biallelic *ATM* loss (frameshift mutation with deletion of the second allele), who initially presented with ataxic gait and T‐cell lymphopenia. (B) Granulomatous skin lesions in a patient with AT due to *ATM* deficiency; histopathologic examination revealed granulomatous inflammation, and microbiological investigations were negative for mycobacterial and fungal pathogens. (C) Scleral telangiectasia in a patient with AT due to biallelic *ATM* deficiency, accompanied by CD4⁺ T‐cell lymphopenia, reflecting characteristic vascular involvement associated with AT. (D) Alopecia areata with complete loss of eyebrows and eyelashes in a patient with immune dysregulation due to biallelic *AIRE* pathogenic variants, consistent with autoimmune‐mediated cutaneous and hair involvement. (E) Norwegian scabies in a patient with AT due to biallelic *ATM* deficiency, accompanied by profound CD4⁺ T‐cell lymphopenia, requiring prolonged systemic antiparasitic treatment and complicated by subsequent lymphoma development. (F) Disseminated granulomatous skin lesions as the initial presenting feature in a patient with AT due to biallelic *ATM* deficiency, accompanied by lymphopenia; granulomatous inflammation was confirmed histologically, and extensive microbiological investigations did not identify an infectious agent. (G) Recurrent herpetic skin lesions in a patient with *NFKB1* deficiency, initially evaluated for hypogammaglobulinemia and recurrent viral infections, consistent with antibody deficiency and impaired antiviral immunity. (H) Recurrent cutaneous abscesses caused by *Staphylococcus aureus* and herpetic skin lesions in a patient with autosomal recessive *DOCK8* deficiency due to biallelic gene deletion, associated with reduced CD3⁺ T‐cell counts; despite normal immunoglobulin levels, the patient showed a favorable clinical response to immunoglobulin replacement therapy, reflecting combined immunodeficiency with susceptibility to bacterial and viral infections. (I) Ecthyma gangrenosum presenting as a necrotic ulcerative skin lesion in a patient with congenital neutropenia due to *HAX1* deficiency, associated with severe neutropenia and recurrent bacterial infections, classically linked to *Pseudomonas aeruginosa*. (J) Perianal abscess and fistula formation in a patient with *IL10* receptor deficiency, initially investigated due to very early‐onset, treatment‐refractory inflammatory bowel disease, consistent with immune dysregulation–associated cutaneous involvement. (K) Molluscum contagiosum and extensive viral warts in a patient with *STK4* deficiency; human papillomavirus infection was confirmed by skin biopsy, and the patient had concomitant refractory eczema, Epstein–Barr virus viremia, and reduced recent thymic emigrant T‐cell levels, reflecting impaired T‐cell–mediated antiviral immunity. AIRE – Autoimmune Regulator, AT – Ataxia–Telangiectasia, ATM – Ataxia–Telangiectasia Mutated, CD3⁺ – Cluster of Differentiation 3 positive T lymphocytes, CD4⁺ – Cluster of Differentiation 4 positive T lymphocytes, DOCK8 – Dedicator of Cytokinesis 8, HAX1 – HS1‐Associated Protein X‐1, HPV – Human Papillomavirus, IL10 – Interleukin‐10, NFKB1 – Nuclear Factor Kappa B Subunit 1.

Additionally, 35 patients (17.7%) exhibited disease‐specific skin manifestations directly related to their underlying IEI diagnosis. These included telangiectasia in ataxia‐telangiectasia (*n* = 27), silvery gray hair and skin dyspigmentation in Griscelli syndrome (*n* = 1) and Chediak‐Higashi syndrome (*n* = 2), palmoplantar keratosis, hyperpigmentation, and nail dystrophy in ectodermal dysplasia with immunodeficiency (*n* = 2), and angioedema in C1 esterase inhibitor deficiency (*n* = 3). These findings were present in all affected patients within these subgroups, underscoring their diagnostic specificity (Supporting Information S1: Table S3). Figure 5 present representative clinical photographs demonstrating selected cutaneous, ocular, and hair findings associated with specific IEI subtypes.

## 4 Discussion

Cutaneous manifestations are among the most frequent and earliest clinical signs of IEIs and often provide critical diagnostic clues [3, 7]. In our cohort, more than half of the patients exhibited cutaneous, ocular and/or hair involvement, a prevalence comparable to reports from regions with similar genetic backgrounds and consanguinity rates, and higher than that described in several other international cohorts [4, 10, 11, 12, 13]. Importantly, cutaneous, ocular and/or hair findings were present at diagnosis in approximately one‐third of patients, reinforcing the role of dermatologic features as early indicators prompting immunologic evaluation.

The prevalence of mucocutaneous, ocular and/or hair involvement varied across IEI subgroups, with the highest rates observed in disorders affecting phagocyte function, combined immunodeficiencies, and syndromic forms of IEI. In contrast, patients with PAD showed the lowest frequency of these manifestations. These patterns suggest an association between impaired cellular immunity and increased susceptibility to cutaneous, ocular, and/or hair manifestations; however, the dominant IEI categories vary across populations, with antibody deficiencies predominating in some cohorts, such as Colombia [11].

Infectious dermatoses represented the most common cutaneous manifestations in our study, with bacterial infections predominating, particularly in phagocytic defects such as congenital neutropenia and CGD. This distribution aligns with findings from multiple international cohorts [12, 13]. Primary immunodeficiencies are associated with an increased risk of Bacille Calmette–Guérin (BCG)‐related complications, particularly in SCID, CGD, MSMD [14, 15]. In countries with routine BCG vaccination, such as the setting of the present study, persistent or severe BCG‐related cutaneous manifestations should prompt consideration of an underlying immunodeficiency consistent with the frecuency observed in our cohort. Notably, fungal infections constituted the second most frequent infectious category in our population, a pattern also reported in selected cohorts [13, 16]. This observation suggests that host immunologic factors—especially defects affecting cellular and humoral immunity—may play a more prominent role than environmental or climatic conditions alone in determining fungal susceptibility [4, 16]. Persistent or recurrent mucocutaneous candidiasis was particularly common among patients with SCID, consistent with its recognized role as an early warning sign of profound immune dysfunction [17]. Candidiasis associated with eczema, a characteristic feature of HIES [12, 17], was also reflected in our cohort, where eczema was observed in 85.8% of patients with HIES and half of these patients presented with concomitant candidiasis, predominantly involving the nails and intertriginous areas. In addition, invasive fungal disease with cutaneous extension underscored the potential severity of fungal infections in patients with profound immune defects.

Viral cutaneous infections were less frequent but clinically significant, including extensive warts, molluscum contagiosum, and recurrent herpetic lesions. Severe viral skin involvement in patients with T‐cell defects, such as STK4 deficiency, highlights the importance of recognizing refractory or extensive viral infections as markers of underlying cellular immune impairment. Parasitic infections, including refractory and Norwegian scabies, were also observed, particularly in patients with ataxia–telangiectasia, emphasizing the risk of atypical and severe parasitic disease in immunodeficient individuals [18].

Eczematous dermatitis represented the second most common category of cutaneous manifestations. When severe, early‐onset, or refractory to standard therapy, eczema may signal an underlying IEI, particularly in the presence of recurrent infections or failure to thrive [19, 20]. In our cohort, eczema was especially prevalent in HIES and was also observed in patients with PAD (especially selective IgA deficiency). The wide variation in reported eczema prevalence across international cohorts likely reflects differences in patient populations, diagnostic criteria, and referral patterns [4, 12, 13, 21].

Disease‐specific cutaneous findings, although less frequent in our cohort, demonstrated high diagnostic value. Telangiectasia in ataxia–telangiectasia, pigmentary abnormalities in Griscelli and Chediak–Higashi syndromes, palmoplantar keratosis and nail dystrophy in ectodermal dysplasia with immunodeficiency, and angioedema in C1 esterase inhibitor deficiency were observed exclusively within their respective subgroups. Granulomatous skin lesions, while rare, were identified as an initial presenting feature in some patients and may represent an important clue to underlying disorders such as CGD or DNA repair defects [10, 20, 22].

The broad spectrum of genetic diagnoses observed in our cohort reflects the marked heterogeneity of IEIs presenting with mucocutaneous, ocular, and hair involvement. While our study was not designed to perform formal genotype–phenotype correlation analyses, certain genetic categories were consistently associated with characteristic clinical patterns. In particular, DNA repair defects, predominantly *ATM* deficiency, were frequently accompanied by telangiectasia and granulomatous skin lesions, whereas defects affecting cellular immunity were more often associated with severe infectious manifestations involving the cutaneous and ocular systems. These observations highlight the clinical utility of integrating genetic classification with careful phenotypic assessment, while underscoring the need for larger, genotype‐focused studies to define precise correlations.

Beyond the cutaneous, ocular and hair manifestations provided additional diagnostic insights. Viral retinitis, keratitis, and conjunctivitis were predominantly observed in patients with severe or combined immunodeficiencies, whereas scleral telangiectasia was characteristic of ataxia–telangiectasia. Hair abnormalities, although infrequent, showed high specificity for certain IEI subtypes, including trichorrhexis nodosa in trichohepatoenteric syndrome and silvery‐gray hair in Griscelli and Chediak–Higashi syndromes. Autoimmune‐mediated alopecia areata was observed in selected disorders of immune dysregulation and autoinflammation, further underscoring the value of integrating ectodermal findings into the diagnostic evaluation.

**Figure 6:**
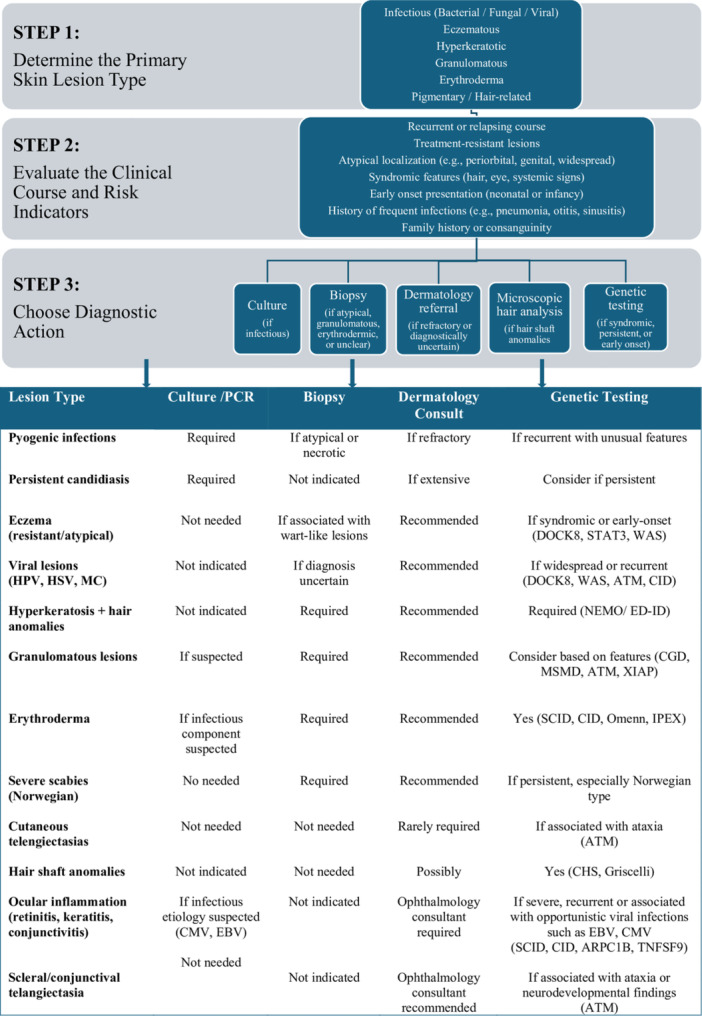
Evaluation Flowchart for IEI‐Associated mucocutaneous, hair or ocular lesions. ARPC1B, Actin‐related protein complex subunit 1B; ATM, Ataxia‐Telangiectasia mutated; CGD, Chronic Granulomatous Disease; CHS, Chediak‐Higashi Syndrome; CID, Combined Immunodeficiency; CMV, Cytomegalovirus; DOCK8, Dedicator of Cytokinesis 8 Deficiency; EBV, Epstein–Barr virüs; ED‐ID, Ectodermal Dysplasia with Immunodeficiency; HPV, Human Papillomavirus; HSV, Herpes Simplex Virus; IPEX, Immune Dysregulation; Polyendocrinopathy; Enteropathy; X‐linked Syndrome; MC, Molluscum Contagiosum; MSMD, Mendelian Susceptibility to Mycobacterial Disease; NEMO, NF‐κB Essential Modulator Deficiency; STAT3, Signal Transducer and Activator of Transcription 3 Deficiency; TNFSF9, Tumor necrosis factor superfamily member 9; WAS, Wiskott‐Aldrich Syndrome; XIAP, X‐linked Inhibitor of Apoptosis Deficiency.

Overall, our findings emphasize the importance of comprehensive dermatologic and ophthalmologic assessment—including cutaneous, ocular, hair, and nail findings—in patients with suspected or confirmed IEI. Recognition of recurrent, atypical, or treatment‐resistant manifestations can facilitate earlier diagnosis and targeted immunologic work‐up, particularly in regions with high consanguinity rates. To support clinical decision‐making, we propose a practical algorithm integrating lesion type, diagnostic yield, and associated immunologic features to guide the use of microbiological studies, biopsy, specialist consultation, and genetic testing (Figure 6).

## 5 Limitations

This study has several limitations. Although a substantial proportion of patients had genetically confirmed diagnoses, comprehensive genotype–phenotype correlation analyses were limited by the marked heterogeneity of IEI subtypes and the absence of uniform, comprehensive molecular testing across the entire cohort. In addition, the retrospective, single‐center design and the relatively small number of patients within certain IEI subgroups may limit the generalizability of the findings. Furthermore, direct comparison with adult IEI cohorts is constrained by the lack of studies that systematically and concurrently evaluate cutaneous, ocular, and hair manifestations within a unified phenotyping framework, as the available adult literature is largely organ‐ or disease‐specific.

## Author Contributions

**Burcu Cil Yılmaz:** conceptualization, writing – original draft, writing – review and editing, visualization, project administration, methodology, investigation, formal analysis, data curation, software. **Sibel Kaplan Sarikavak:** data curation, investigation, writing – review and editing. **Sezin Naiboglu:** writing – review and editing, resources, project administration. **Gulsah Kalay:** resources, writing – review and editing. **Erkan Cakmak:** writing – review and editing, validation, formal analysis. **Ozge Turkyilmaz Ucar:** validation, writing – review and editing. **Selami Ulas:** writing – review and editing, resources. **Nermin Kapci:** project administration, writing – review and editing. **Pinar Gokmirza:** writing – review and editing, validation, supervision. **Cigdem Aydogmus:** supervision, conceptualization, methodology, writing – review and editing, writing – original draft.

## Funding

The authors received no specific funding for this work.

## Conflicts of Interest

The authors declare no conflicts of interest.

## Supporting information


**Supplementary Table 1:** Genetic Distribution and Subgroups of Patients with Inborn Errors of Immunity. **Supplementary Table 2:** Distribution of mucocutaneous, ocular, and hair findings across IEI subgroups. **Supplementary Table 3:** Disease‐specific mucocutaneous, hair and ocular findings in IEI patients. **Supplementary Figure 1:** Heatmap of mucocutaneous, ocular, and hair findings across IEI groups (% relative to the entire IEI cohort). **Supplementary Figure 2:** Heatmap of mucocutaneous, ocular, and hair findings across IEI groups (% relative to patients with these manifestations). **Supplementary Figure 3:** Heatmap of mucocutaneous, ocular, and hair findings across IEI subgroups (% relative to the entire IEI cohort). **Supplementary Figure 4:** Heatmap of mucocutaneous, ocular, and hair findings across IEI subgroups (% relative to patients with these manifestations).

## Data Availability

Data available on request due to privacy/ethical restrictions.
