# Supplementary material for: Clinical Spectrum of Cutaneous, Ocular, and Hair Manifestations in Patients With Inborn Errors of Immunity: Insights From a Single Center in Turkey
Source: Immun Inflamm Dis. 2026 Feb 25;14(2):e70384. doi: 10.1002/iid3.70384 (PMC12933409; doi:10.1002/iid3.70384)
Supplement: Supplementary file 1 — Supplementary Table 1: Genetic Distribution and Subgroups of Patients with Inborn Errors of Immunity. Supplementary Table 2: Distribution of mucocutaneous, ocular, and hair findings across IEI subgroups. Supplementary Table 3: Disease‐specific mucocutaneous, hair and ocular findings in IEI patients. Supplementary Figure 1: Heatmap of mucocutaneous, ocular, and hair findings across IEI groups (% relative to the entire IEI cohort). Supplementary Figure 2: Heatmap of mucocutaneous, ocular, and hair findings across IEI groups (% relative to patients with these manifestations). Supplementary Figure 3: Heatmap of mucocutaneous, ocular, and hair findings across IEI subgroups (% relative to the entire IEI cohort). Supplementary Figure 4: Heatmap of mucocutaneous, ocular, and hair findings across IEI subgroups (% relative to patients with these manifestations). [file IID3-14-e70384-s001.docx]

| **IEI Category (IUIS Classification)** | **Subgroup / Phenotype** | **Total Patients**  **(n)** | **Genetic Defects Identified (n)** |
| --- | --- | --- | --- |
| **Immunodeficiencies affecting cellular and humoral immunity** | Severe Combined Immunodeficiency | 23 | RAG1-RAG2 (11), IL2RG (2), JAK3 (2), ADA (2), Artemis (1), Cernunnos (1), Undiagnosed* (4) |
|  | Combined Immunodeficiency | 17 | CD40L (5), DOCK8 (5), MALT1 (2), MHC2 (2), ARPC1B (1), FCHO (1), STK4 (1) |
| **Combined immunodeficiencies with associated or syndromic features** | DNA repair defects | 27 | ATM (27) |
|  | Thymic defects | 9 | 22q11.2 deletion (9) |
|  | Hyper IgE syndromes | 7 | STAT3 (3), PGM-3 (2), SPINK5 (1), IL6ST (1) |
|  | Anhidrotic ectodermal dysplasia | 2 | IKBKG (2) |
|  | Others | 9 | PNP (4), TTC37 (3), SP110/VODI (1), RBCK1/HOIL1 (1) |
| **Predominantly antibody deficiencies** | Agammaglobulinemia | 5 | BTK (4), IGLL1 (1) |
|  | CVID phenotype | 8 | TNFRSF13B (4), NFKB1 (2), SEC61A1 (1), SH3KBP1 (1) |
|  | Isotype/Light chain/ Functional B cell defects | 22 | Selective IgA deficiency (17), Isolated IgG3 deficiency (2), Combined IgA & IgG3 deficiency (3) |
| **Congenital defects of phagocyte number or function** | Congenital neutropenias | 23 | HAX1 (17), ELANE (2), USB1 (2), SRP54 (1), JAGN (1) |
|  | Defects of motility | 4 | LAD type 1 (4) |
|  | Defects of respiratory burst | 12 | CGD: CYBB (6), NCF1 (6) |
| **Diseases of immune dysregulation** | - | 10 | LYST (2), FAS (2), RAB27A (1), IL10R (1), AIRE (1), CASP10 (1), LRBA (1), TNFRSF9 (1) |
| **Defects in intrinsic and innate immunity** | - | 7 | IL12RB1 (2), STAT1 (1), IRAK4 (2), GATA2 (1), MYD88 (1) |
| **Autoinflammatory disorders** | - | 7 | ADA2 (4), PLCG2 (2), MEFV/STAT4 (1) |
| **Complement deficiencies** | - | 3 | SERPING1 (3) |
| **Bone marrow failure** | - | 1 | RTEL1 (1) |
| **Phenocopies of IEI** | - | 2 | Unproven CMC (2) |

**Supplementary Table 1.** Genetic Distribution and Subgroups of Patients with Inborn Errors of Immunity

***Note:** 4 patients with SCID underwent Hematopoietic Stem Cell Transplantation without a definitive genetic diagnosis.

**ADA:** Adenosine Deaminase, **ADA2:** Adenosine Deaminase 2, **AIRE:** Autoimmune Regulator, **ARPC1B:** Actin Related Protein Complex Subunit 1B, **ATM:** Ataxia-Telangiectasia Mutated, **BTK:** Bruton Tyrosine Kinase, **CASP10:** Caspase 10, **CD40L:** CD40 Ligand, **CGD:** Chronic Granulomatous Disease, **CID:** Combined Immunodeficiency, **CMC:** Chronic Mucocutaneous Candidiasis, **CVID:** Common Variable Immunodeficiency, **CYBB:** Cytochrome B-245 Beta Chain, **DOCK8:** Dedicator of Cytokinesis 8, **ELANE:** Elastase, Neutrophil Expressed, **FAS:** Fas Cell Surface Death Receptor, **FCHO1:** FCH Domain Only 1, **GATA2:** GATA Binding Protein 2, **HAX1:** HCLS1 Associated X 1, **HOIL1:** RanBP-Type And C3HC4-Type Zinc Finger Containing 1 (also known as RBCK1), **HSCT:** Hematopoietic Stem Cell Transplantation, **IEI:** Inborn Errors of Immunity, **IGLL1:** Immunoglobulin Lambda Like Polypeptide 1, **IKBKG:** Inhibitor of Nuclear Factor Kappa B Kinase Regulatory Subunit Gamma, **IL2RG:** Interleukin 2 Receptor Subunit Gamma, **IL6ST:** Interleukin 6 Cytokine Family Signal Transducer, **IL10R:** Interleukin 10 Receptor, **IL12RB1:** Interleukin 12 Receptor Subunit Beta 1, **IRAK4:** Interleukin 1 Receptor Associated Kinase 4, **JAGN1:** Jagunal Homolog 1, **JAK3:** Janus Kinase 3, **LAD:** Leukocyte Adhesion Deficiency, **LRBA:** LPS Responsive Beige-Like Anchor Protein, **LYST:** Lysosomal Trafficking Regulator, **MALT1:** MALT1 Paracaspase, **MEFV:** MEterranian FeVer (Pyrin), **MHC2:** Major Histocompatibility Complex Class II, **MYD88:** Myeloid Differentiation Primary Response 88, **NCF1:** Neutrophil Cytosolic Factor 1, **NFKB1:** Nuclear Factor Kappa B Subunit 1, **PGM3:** Phosphoglucomutase 3, **PLCG2:** Phospholipase C Gamma 2, **PNP:** Purine Nucleoside Phosphorylase, **RAG1/2:** Recombination Activating Gene ½, **RBCK1:** RanBP-Type And C3HC4-Type Zinc Finger Containing 1, **RTEL1:** Regulator of Telomere Elongation Helicase 1, **SCID:** Severe Combined Immunodeficiency, **SEC61A1:** SEC61 Translocon Alpha 1 Subunit, **SERPING1:** Serpin Family G Member 1, **SH3KBP1:** SH3 Domain Containing Kinase Binding Protein 1, **SP110:** SP110 Nuclear Body Protein, **SPINK5:** Serine Peptidase Inhibitor Kazal Type 5, **SRP54:** Signal Recognition Particle 54, **STAT1/3/4:** Signal Transducer and Activator of Transcription 1/3/4, **STK4:** Serine/Threonine Kinase 4, **TNFRSF9/13B:** TNF Receptor Superfamily Member 9/13B, **TTC37:** Tetratricopeptide Repeat Domain 37, **USB1:** U6 snRNA Biogenesis Phosphodiesterase 1, **VODI:** Veno-occlusive Disease with Immunodeficiency

| **IEI Category** | Total N of patients | With  skin ManifestationsN | Skin infections  N | Eczema  N | Non-eczematous  immune-  allergic  findings  N | Spesific to an IEI  N | Other  N | Hair  Findings  N | Eye  Findings  N |
| --- | --- | --- | --- | --- | --- | --- | --- | --- | --- |
| Immunodeficiencies affecting cellular and humoral immunity   - SCID - CID | 61  35  26 | 40  23  17 | 31  17  14 | 14  6  8 | 1  1  - | - | 2  1  1 | - | 3  2  1 |
| Combined immunodeficiencies with associated or syndromic features   - DNA repair defects - Thymic defects with congenital anomalies - Hyper IgE syndromes - Anhidrotic ectodermodysplasia-Immunodeficiency - Other defects | 93  27  42  8  2  14 | 54  27  9  7  2  9 | 26  7  8  5  -  6 | 17  4  1  **6**  2  4 | 2  -  -  -  2  - | 29  27  -  -  2  - | 3  2  -  1  -  - | 3  -  -  -  -  3 | 27  27  -  -  -  - |
| Predominantly antibody deficiencies   - Severe reduction, agamaglobulinemia - Severe reduction in at least 2 serum Ig ısotypes, CVID phenotype - Isotype, Light chain, Functional deficiencies with normal B cells | 145  8  15  122 | 35  5  8  22 | 30  3  7  20 | 26  2  2  22 | - | - | 1  1  -  - | - | - |
| Congenital defects of phagocyte number or function   - Congenital neutropenias - Defects of motility - Defects of respiratory burst | 44  25  4  15 | 39  23  4  12 | 39  23  4  12 | 1  -  1  - | - | - | 2  1  -  1 | - | - |
| Diseases of immune dysregulation | 17 | 10 | 2 | 1 | 3 | 3 | - | 4 | 1 |
| Defects in intrinsic and innate immunity | 7 | 7 | 6 | 1 | - | - | 2 | - | - |
| Autoinflammatory disorders | 12 | 7 | 7 | - | - | - | - | 1 | - |
| Complement deficiencies | 3 | 3 | - | - | 3 | 3 | - | - | - |
| Bone narrow failure | 2 | 1 | 1 | - | - | - | 1 | - | - |
| Phenocopies of IEI associated with autoantibodies or somatic variants | 2 | 2 | 2 | - | - | - | - | - | - |
| Total | 386 | 198 | 142 | 60 | 9 | 35 | 11 | 8 | 31 |

**Supplementary Table 2.** Distribution of mucocutaneous, ocular, and hair findings across IEI subgroups

**Note: CID**: combined immunodeficiency, **CVID**: common variable immune deficiency, **IEI:** inborn errors of immunity, I**g**: immunoglobulin, **SCID**: severe combined immunodeficiency.

| Diagnosis – N | Spesific Manifestations – N (%) |
| --- | --- |
| Ataxia-telangiectasia - 27 | Telangiectasia – 27 (%100) |
| Chediak–Higashi syndrome - 2 | Silvery gray hair + skin hypopigmentation - 2 (%100) |
| Griscelli syndrome - 1 | Silvery gray hair + skin dyspigmentation -1(%100) |
| Anhidrotic ectodermodysplasia with immunodeficiency - 2 | Palmoplantar keratosis and hyperpigmentation -2 (%100) |
| C1 esterase inhibitor deficiency - 3 | Angioedema -3 (%100) |
|  |  |

**Supplementary Table 3**. Disease-specific mucocutaneous, hair and ocular findings in IEI patients

**Note: C1:** Complement 1

**Supplementary Figure 1.** Heatmap of mucocutaneous, ocular, and hair findings across IEI groups (% relative to the entire IEI cohort)


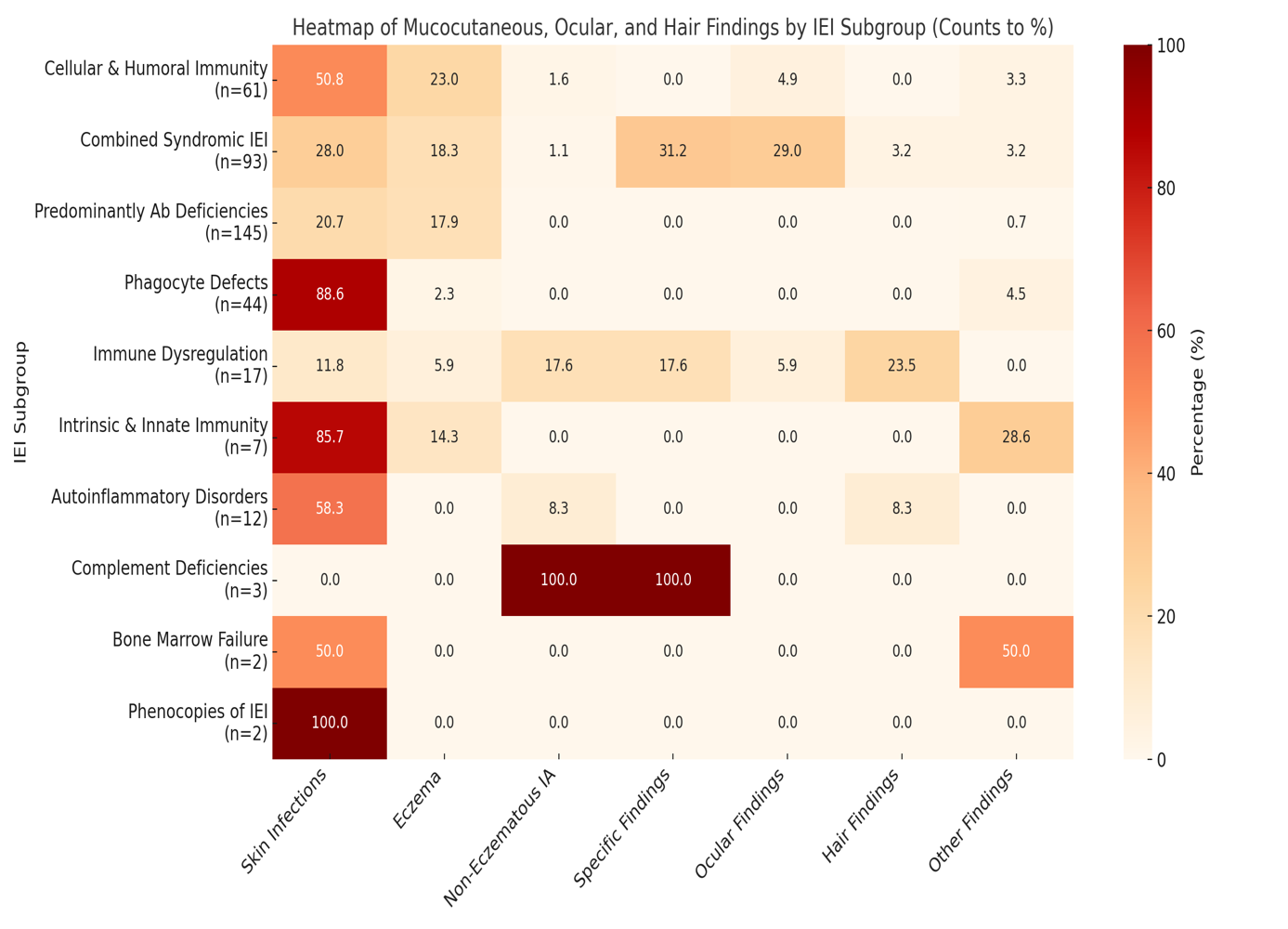


Distribution of cutaneous, ocular, and hair findings among IEI groups. Values represent percentages relative to the total number of patients in each IEI category. Color intensity corresponds to frequency.

(Ab: antibody IA: immune-allergic, IEI: inborn errors of immunity)

**Supplementary Figure 2.** Heatmap of mucocutaneous, ocular, and hair findings across IEI groups (% relative to patients with these manifestations).


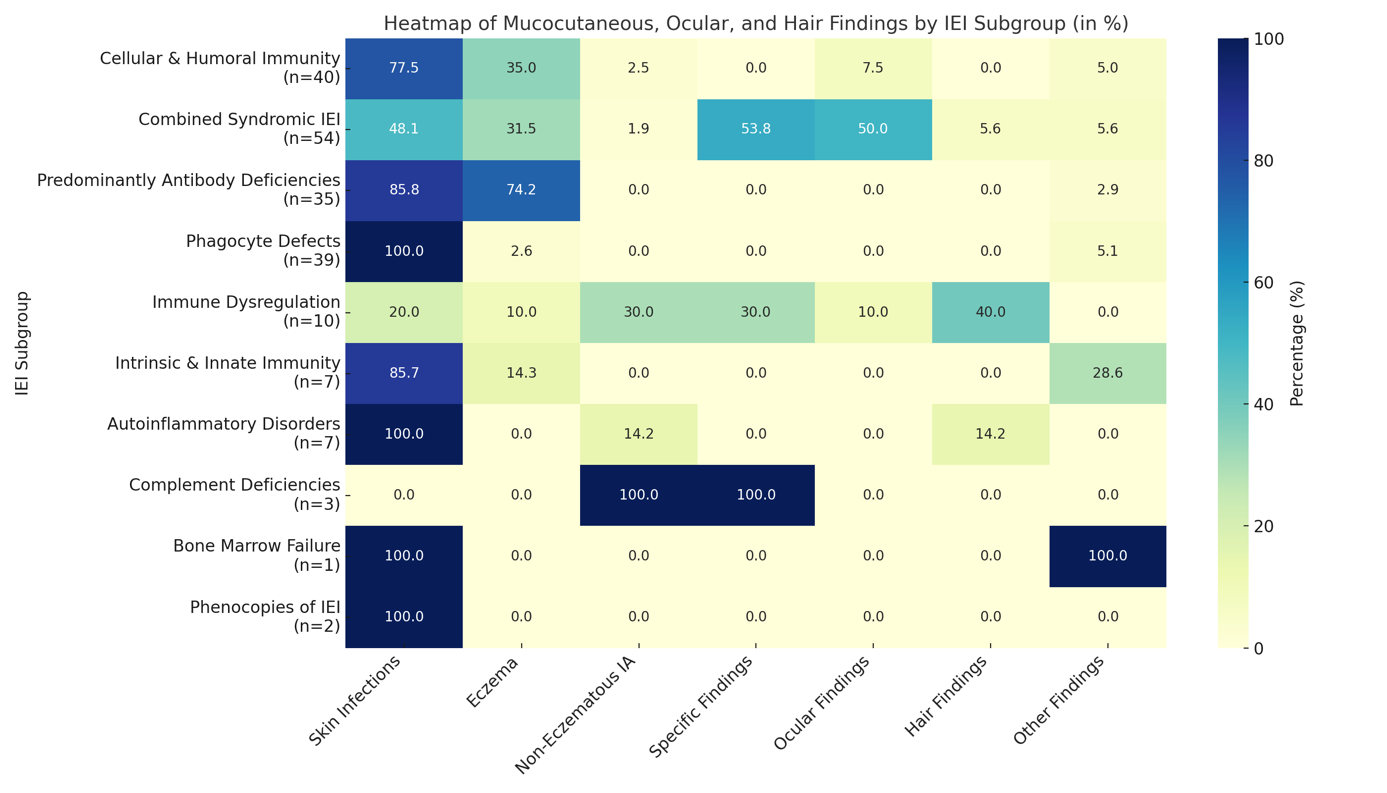


Percent distribution of cutaneous, ocular, and hair findings among IEI groups, limited to patients with mucocutaneous, ocular, or hair involvement (n = 198). Color intensity corresponds to frequency.

(IA: immune-allergic, IEI: inborn errors of immunity)

**Supplementary Figure 3.** Heatmap of mucocutaneous, ocular, and hair findings across IEI subgroups (% relative to the entire IEI cohort)


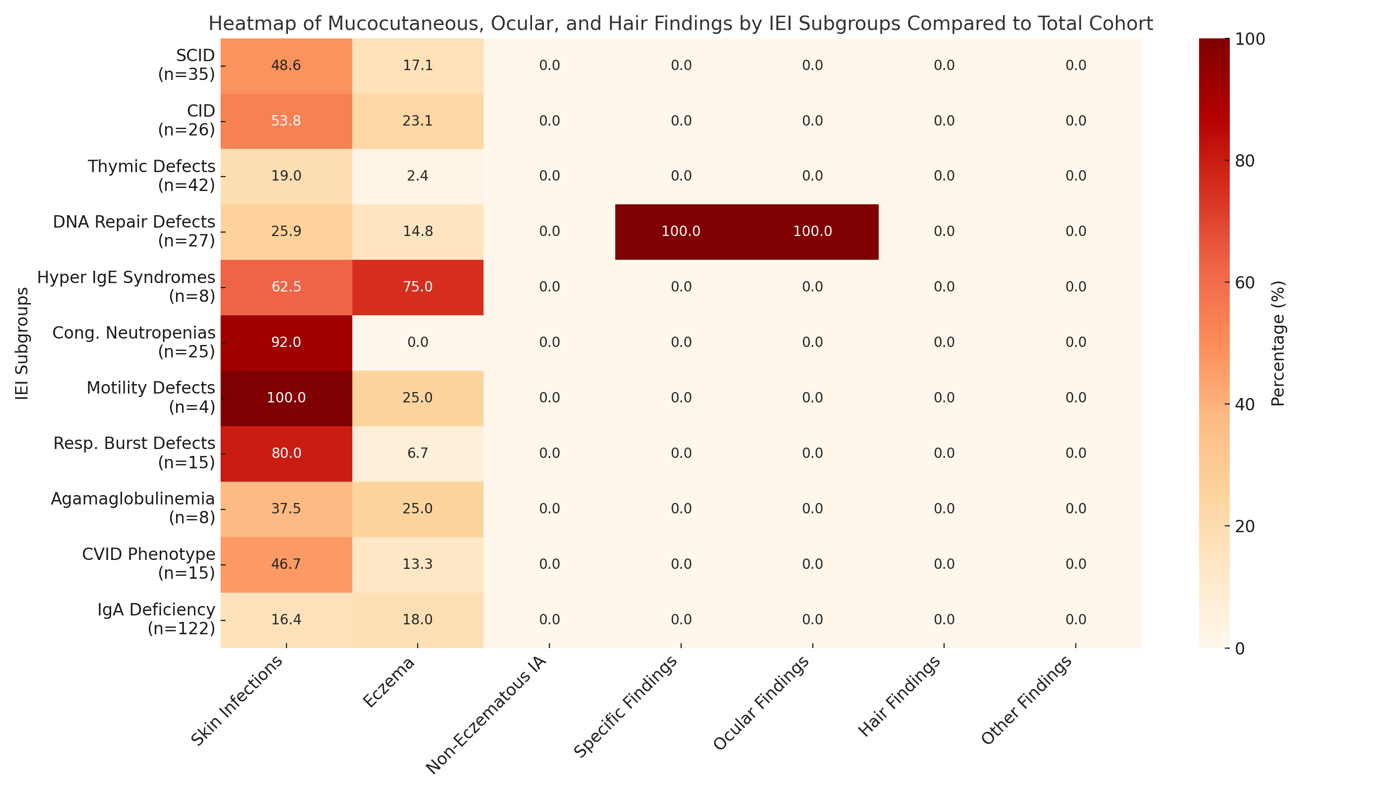


Distribution of cutaneous, ocular, and hair findings among IEI subgroups. Values represent percentages relative to the total number of patients in each IEI category. Color intensity corresponds to frequency.

(Cong: cengenital, CID: combined immunodeficiency, CVID: common variable immune deficiency, IA: immune-allergic,

IEI: inborn errors of immunity, Resp: Respiratory, SCID: severe combined immunodeficiency)

**Supplementary Figure 4.** Heatmap of mucocutaneous, ocular, and hair findings across IEI subgroups (% relative to patients with these manifestations).


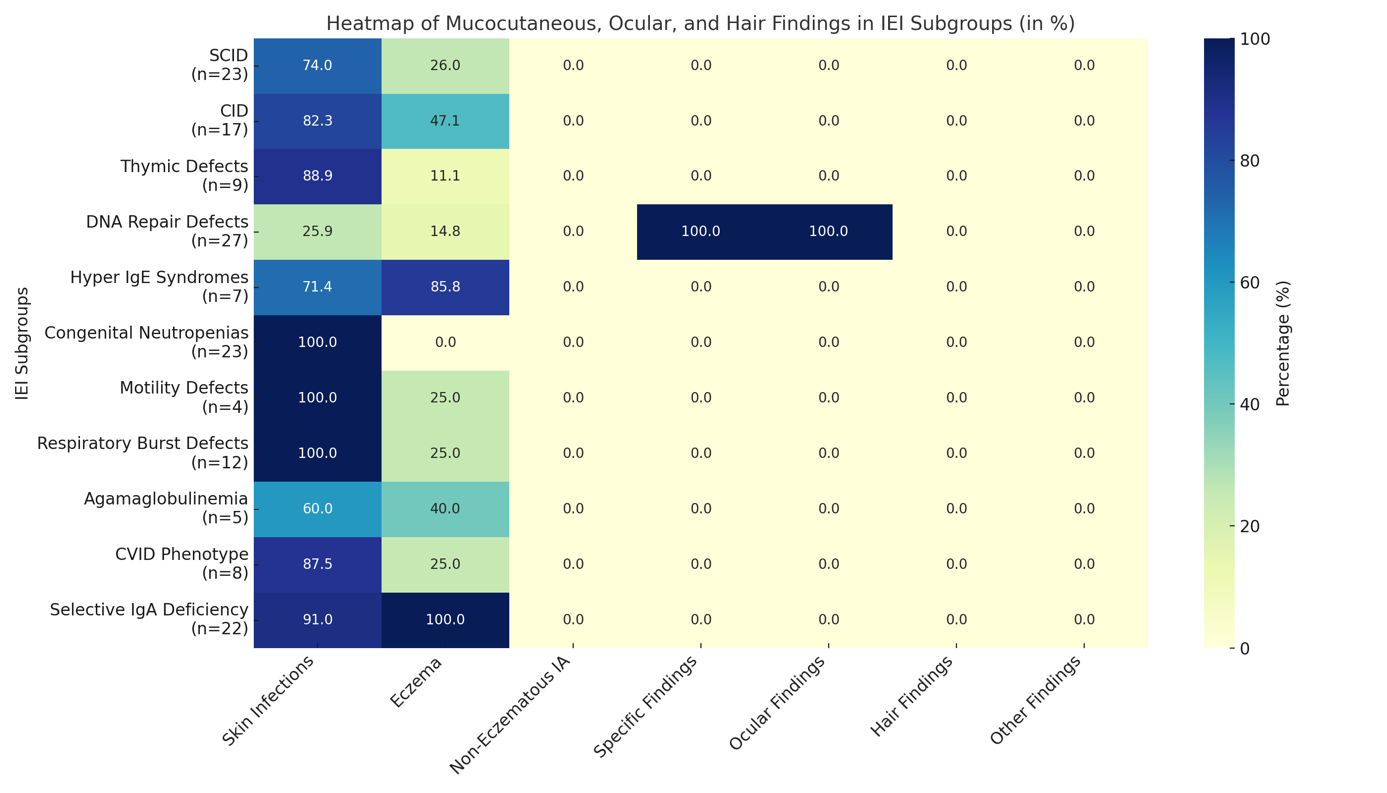


This heatmap presents the percentage distribution of mucocutaneous, ocular, and hair findings across specific IEI subgroups (e.g., SCID, CID, Hyper IgE syndrome, Selective IgA deficiency) Percentages are calculated relative to the number of patients within each subgroup who exhibited these findings. Color intensity corresponds to frequency.

(CID: combined immunodeficiency, CVID: common variable immune deficiency, IA: immune-allergic, IEI: inborn errors of immunity, SCID: severe combined immunodeficiency)
